# Uncovering a novel component in the cyanobacterial carbon concentration mechanism

**DOI:** 10.1093/plphys/kiag212

**Published:** 2026-04-13

**Authors:** Rose McNelly

**Affiliations:** Assistant Features Editor, Plant Physiology, American Society of Plant Biologists; John Innes Centre, Norwich Research Park, Norwich NR4 7UH, United Kingdom

Cyanobacteria are key players in global biogeochemical cycles as they are responsible for at least 25% of oceanic carbon fixation (Sánchez-Baracaldo et al. 2022). Fixation of carbon dioxide (CO_2_) takes place in the Calvin cycle catalyzed by Rubisco (ribulose-1,5-bisphosphate carboxylase/oxygenase). Yet Rubisco faces many difficulties in today's atmosphere—it has low specificity for CO_2_ and, due to higher concentrations of atmospheric oxygen than carbon dioxide, frequently catalyzes an undesired reaction with oxygen (Lechno-Yossef et al. 2020). Cyanobacteria face additional challenges, as aquatic environments have less accessible forms of carbon, mainly HCO_3_^−^ as opposed to CO_2_, and the rate of diffusion is slower (Nguyen et al. 2025). Hence, the supply of inorganic carbon (Ci) in water is lower than that on land, which further limits Rubisco.

To overcome Rubisco's limitations, cyanobacteria have evolved a carbon concentrating mechanism (CCM). The CCM relies on cytoplasmic accumulation of HCO_3_^−^, which diffuses into Rubisco-containing protein compartments, called carboxysomes. Inside carboxysomes HCO_3_^−^ is converted to CO_2_ by carbonic anhydrases, resulting in high concentrations of CO_2_ around Rubisco, which improves the efficiency of carbon fixation (Nguyen et al. 2025). Uptake of Ci is a key step for the cyanobacterial CCM, so it is unsurprising that there are at least 5 uptake systems (Rottet et al. 2021). One is the sodium-dependent bicarbonate transporter A (SbtA) that is induced under low CO_2_ and has a high affinity for HCO_3_^−^ (Shibata et al. 2002). For many years the regulation of SbtA remained enigmatic until the identification of SbtB that may inhibit SbtA-mediated HCO_3_^−^ transport and prevent HCO_3_^−^ leakage during the dark (Du et al. 2014; Haffner et al. 2023). Beyond SbtB no additional proteins are known to be involved in SbtA-dependent transport, so identification of additional regulators is of great interest.

In a recent article in *Plant Physiology*, Walke et al. (2026) identify a previously unannotated small open reading frame (ORF) upstream of *SbtA* and *SbtB* in the model cyanobacterium *Synechocystis* sp. PCC 6803, which they call *SbtC*. Using northern blotting, they demonstrated that *SbtC* is transcribed under low CO_2_ conditions. To confirm whether the transcript produced a functional protein, the gDNA was fused to a FLAG-tag encoding sequence and placed under a copper response promoter in *Synechocystis*. When copper was supplied, a FLAG-tag protein was detected via western blotting. Together, these results suggested that *SbtC* is responsive to low CO_2_ and produces a protein.

The authors proceeded in generating a Δ*sbtC* mutant in *Synechocystis* using homologous recombination with a gentamicin resistance cassette. Generally, Δs*btC* mutants had no growth phenotype except under diurnal light and low CO_2_ conditions when growth was slightly, but not significantly, reduced. This phenotype resembled that of a *sbtB* mutant (Selim et al. 2021). The relatively minor growth phenotypes are unsurprising given the redundancy in cyanobacterial Ci uptake mechanisms (Rottet et al. 2021). Whether the Δ*sbtC* mutation, or indeed a *sbtB* mutation, would have a stronger effect in noncontrolled conditions has not been tested.

To test whether SbtC influences Ci uptake, the authors utilized the Δ5 mutant. The Δ5 mutant lacks all Ci uptake systems and is a robust background to use for validating putative Ci uptake mechanisms (Xu et al. 2008). The *SbtABC* genes were re-introduced in the Δ5 mutant, and *SbtB* or *SbtC* was knocked out independently (Fig. 1). Comparative analysis of these strains revealed that SbtC inhibits SbtA-dependent Ci transport, as strains without *SbtC* have greater Ci uptake than strains expressing *SbtC* (Fig. 1a). Both SbtB and SbtC minimized Ci leakage via SbtA during periods of darkness, as strains lacking either protein had significantly greater Ci leakage (Fig. 1b). The role of SbtB in preventing leakage was unsurprising, as it had been hypothesized that SbtB plugs SbtA to prevent Ci loss (Haffner et al. 2023). How SbtC fits into this model is unclear, but perhaps it may stabilize the SbtA-SbtB interaction.

**Figure 1:**
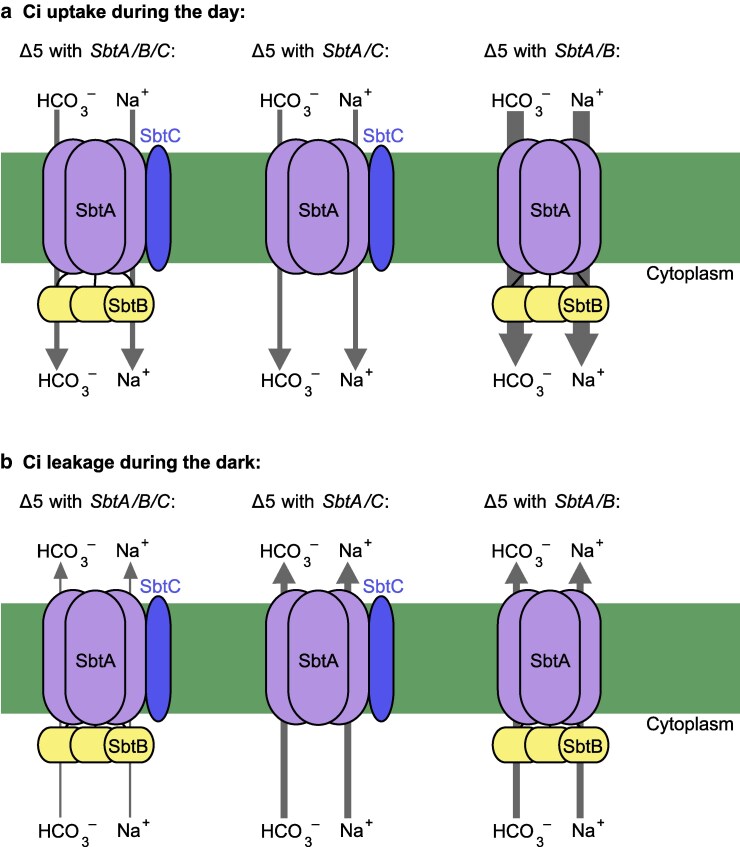
The role of SbtC was deciphered using the Δ5 mutant expressing different combinations of *SbtA/B/C*. The Δ5 mutant lacks all Ci uptake systems. The *SbtABC* genes were re-introduced in the Δ5 mutant (called Δ5 with *SbtA/B/C*), and *SbtB* or *SbtC* was knocked out independently, called Δ5 with *SbtA/C* and Δ5 with *SbtA/B*, respectively. **a)** In the light SbtA/B/C takes up Ci into the cytoplasm, when SbtC is absent there is greater Ci uptake. **b)** In the dark there is leakage of Ci out of the cytoplasm via SbtA/B/C; this leakage is enhanced when either SbtB or SbtC is absent. In (a) and (b) Ci flux is represented by arrow size and width.

SbtA is membrane localized (Fang et al. 2021), and computational analysis suggested that SbtC may have a transmembrane helix. Therefore, the authors proposed that SbtC might stabilize SbtA by direct association in the membrane. To investigate if SbtC forms a complex with SbtA/B, membrane protein complexes from a *Synechocystis* strain expressing FLAG-tagged SbtC were isolated and were analyzed via blue native PAGE and western blotting. SbtA, SbtC, and SbtB proteins co-migrated, which suggested they are in the same complex. Based on expected protein sizes, the authors propose a ternary complex consisting of a SbtA trimer, SbtB trimer, and a SbtC monomer. Additional analysis is required to confirm these proteins directly interact with each other, as protein-protein interaction experiments were not conducted. Furthermore, employing structural techniques to determine the precise conformation of these proteins could provide insights into the molecular mechanism of SbtC action, as similar strategies have been used to successfully elucidate the role of SbtB (Fang et al. 2021).

All experiments conducted here were performed with *Synechocystis* sp. PCC 6803. Curiously, when the authors looked beyond this species, they found that not all cyanobacteria possess a *SbtC* homolog, suggesting that SbtC is not essential. Analysis of the presence/absence of *SbtC* revealed no correlation between *SbtC*'s presence and either phylogeny or cyanobacterial lifestyle. The lack of correlation was surprising and raises several interesting questions. First, is there a selection pressure driving *SbtC* retention in certain species, and if so what is it? Second, does *SbtC* always perform the same role, or does its function vary according to factors, such as the type of environment the cyanobacteria inhabit? And third, in species that do not have *SbtC*, is there an alternative protein that performs its function?

In summary, this work by Walke and colleagues identifies a novel component of the SbtA/B Ci uptake system. Their study identifies the *SbtC* ORF that produces a small protein and affects Ci transport, minimizes Ci leakage and may stabilize the SbtA/B complex. These findings raise several important questions. For example, how is SbtC regulated and expressed in response to Ci limitation? Does SbtC have further unelucidated roles in the cyanobacterial CCM? And more broadly, how many other unannotated ORFs are also producing important proteins for the cyanobacterial CCM or other processes? If anything, this manuscript teaches us the need to revisit genome annotation and ORFs that may have previously been overlooked.

## Related articles in *Plant Physiology*:

- Xin et al. (2026) described the acetylation of a cyanobacterial Ci uptake protein, ChpX, and how this modification is necessary for efficient CO_2_ uptake.
- Sun et al. (2024) deciphered the structural organization of RuBisCo in the carboxysome of *Synechococcus elongatus* PCC7942.

## Data Availability

No new data were generated or analyzed in support of this research.
